# Cast Construction for Artificial Dentures

**Published:** 1921-06

**Authors:** I. Lester Furnas


### CAST CONSTRUCTION FOR ARTIFICIAL DENTURES

BY I. LESTER FURNAS, D.D.S.

I did not choose this subject because the cast or the technic of its construction is the most important step in artificial denture work, for, as I see it, there is no one step that is all-important or stands out in prominence above all others. It is the old story of a chain being no stronger than its weakest link; but I choose it for two main reasons:

First, because I believe a large percentage of the failures in full denture work are due to improperly constructed casts and the abuse of properly constructed casts.

Second, because the technic of construction is such that the average dentist without effort or intention is very likely to slight certain steps which will render an otherwise perfectly good cast almost useless.

It is not within my province in this short paper to discuss any of the merits or demerits of the many different methods of impression-taking; but, in passing this subject, let me say that the advent of the muscle-trimmed impression, whether it be of plaster of paris or modeling compound, has brought with a marked change in our cast construction.

What has been the principal cause, then, for failure as regards the cast?

Paramount among these reasons I should say was the inability of the materials used in its construction to withstand the forces of compression to which the average cast is generally subjected.

The manufacturer of the various cast materials has sacrificed compression in order to overcome expansion and contraction.

Next in importance, I should say, should be placed the fact that the average dentist does not possess a thorough knowledge of his cast materials, or if he possesses it, does not exercise it.

How many of you gentlemen here know just what you are using for your casts?

What constitutes a scientific mix?

What tests have you made upon it for compression?

When is its compression greatest, before or after the sweating stage?

What is its working period?

What do you know about its expansion and contraction?

What pressure do you apply to the face of your cast during the closing of your flask?

To what extent does your cast disintegrate during the process of vulcanization?

At what temperature does this disintegration take place?

Is it before or after the flow of rubber in the flask has stopped?

I ask you these few questions in all seriousness; and then will you not agree with me that so important a thing as our cast, over which we are to fashion our dentures for adaptation, and constructed of materials so fallible, should receive more serious consideration than most of us have given it in the past?

It will not be practicable nor possible for me to attempt to give you here a lengthy review of all cast materials.

They all have their advantages and disadvantages. There is no one material that can be considered as ideal.

Having it understood, then, that I am not recommending over and above all others any particular manufacturers' product, may I not outline a technic that I believe to be highly commendable, using for my cast the material known as Spence plaster compound?

Spence plaster compound when properly mixed has a crushing resistance of 1000 pounds per square inch, 24 hours after mixing, and 1800 pounds per square inch, 12 hours after mixing. Notice, please, that there is a difference of about 800 pounds before and after the mix has gone through the sweating stage. This should tell us that our Spence plaster compound casts should be used, if possible, before they have gone through this sweating stage.

The deterioration of Spence plaster compound casts during the process of vulcanization begins at approximately 300 degrees Fahrenheit, or at about the point where the flow of most rubbers in the flask ceases. This is very desirable because it is very important that our matrix should retain its shape until our denture is fully formed.

This disintegration advances with he heat up to 340 degrees Fahrenheit, at which time it has reached a disintegration point of about sixty per cent.

I have been able to register about two one-thousandths of an inch contraction in Spence plaster compound, but never any expansion; however, in my estimation this change is so small that it is of very minor importance.

Inasmuch, then, as compression is one of the chief requisites of a cast material, and as Spence plaster compound when properly mixed stands very high in compression, and inasmuch as it retains this characteristic after the flask is placed in the vulcanizer and the heat applied to a point of temperature past the rubber flow, then I believe you must agree that it is a good cast material.

There are few men who realize the enormous pressure applied on the face of a cast in closing a flask. Dr.

Prothero, of Chicago, earned the credit for working this out years ago. He found that by using fifty pounds pressure as an average on the end of a four-inch ordinary flask wrench, with a one-to-twenty-pitch ordinary flask bolt, that the face of the cast was subjected to the almost unbelievably terrific strain of over *four tons per square inch;* to be exact it was 8333 pounds plus.

Does this one thing alone explain to any of you men why some of your dentures do not meet all your expectations as regards adaptation and antagonization?

Spring pressure for flask closing is correcting this trouble to a marked degree, but even yet we must exercise more care in our flask closing.

The technic for constructing casts is simple and to most of the profession old.

The impression is imbedded in a heavy mix of plaster of Paris which is termed the foundation reinforcement. This should be about one-eighth-inch thick at its thinnest place, and averaging about one-quarter-inch in thickness. This should be trimmed so that its edges will extend laterally and at right angles to the margins of the impression about one-eighth of an inch.

Next is added a layer of modeling clay which is carried to the turn established by the muscle-trimming and at right angles.

This modeling clay is much better for this work than plaster of Paris because it has no expansion or contraction and is much more easily adapted to the borders of the turn on the impression, and eliminates the danger of breaking frail margins.

Around this reinforcement is then placed a strip of paraffin wax and sealed at the base of the reinforcement so as to be water-tight. This wax border is next trimmed for height. This is easily determined by placing a straight edge from border to border and trimming so that the thinnest part of the cast will be at least one-quarter-inch thick. This wax border may be still further reinforced with plaster of Paris if the wax is very thin. In place of using the paraffin wax for this part of the boxing some men use waxed linen strips, tea-lead strips, rubber-tube banding, cardboard, and many similar things; but I believe that the method given is much better for many reasons.

The entire boxing and impression is now coated with a twenty per cent. solution of shellac in alcohol (filtered). As soon as this is dry, paint with a fifteen per cent. solution of sandarac in alcohol (filtered). When this coating has thoroughly hardened, fill with soapsuds, using a heavy laundry soap in preference to some of the lighter toilet soaps. This soap is then immediately flushed out, but a sufficient amount will remain to act as a separating medium between the cast and the separating mediums proper, and preventing them from sticking to the face of the cast.

The boxing is now filled with water and permitted to stand for from five to ten minutes. This water is now poured into the plaster bowl and will be found to be about the correct amount to use for the proper mix of Spence plaster compound for this particular case.

The Spence plaster compound is now added to this water in sufficient quantity to make a heavy dry mix. This is kneaded in the soft rubber plaster bowl, using the hands in preference to a plaster spatula. When the mix attains a heavy, putty-like consistency it is taken from the plaster bowl and kneaded in the hands, using a rotating or sliding movement to insure the breaking up of any lumps of the material, and insuring a smooth even mix. When it is thoroughly mixed, a small piece about the size of a hickory-nut is pressed in the hands to expel all the air and then placed in the center of the impression. The case is then jarred or beaten up and down on the laboratory bench, table, or something similar, until the Spence plaster compound spreads and fills all the fine lines and details of the impression. More Spence plaster compound is added until the boxing is filled. There are many special lathe devices used in jarring this Spence plaster compound mix into place; but the simple method of beating it down as I have described has been much more satisfactory in my hands.

The cast may be separated in from three to five hours; however, whenever possible it is advisable to let it set longer or over night if convenient.—*Dental Summary*, May, 1921.

